# Early uptake of HIV counseling and testing among pregnant women at different levels of health facilities - experiences from a community-based study in Northern Vietnam

**DOI:** 10.1186/1472-6963-11-29

**Published:** 2011-02-07

**Authors:** Nguyễn Thị Thúy Hạnh, Tine Gammeltoft, Vibeke Rasch

**Affiliations:** 1 Department of Population, Institute of Preventive Medicine and Public Health, Hanoi Medical University, No.1 Ton That Tung Street, Khuong Thuong, Dong Da, Hanoi, Vietnam; 2 Department of Anthropology, University of Copenhagen, Øster Farimagsgade 5, DK-1353 Copenhagen K, Denmark; 3 Department of International Health, Immunology and Microbiology, Faculty of Health Sciences, University of Copenhagen, Øster Farimagsgade 5, DK-1014 Copenhagen, Denmark; 4 Department of Obstetrics and Gynaecology, Odense University Hospital, 5000 Odense C, Denmark

## Abstract

**Background:**

HIV counselling and testing for pregnant women is a key factor for successful prevention of mother to child transmission of HIV. Women's access to testing can be improved by scaling up the distribution of this service at all levels of health facilities. However, this strategy will only be effective if pregnant women are tested early and provided enough counselling.

**Objective:**

To assess early uptake of HIV testing and the provision of HIV counselling among pregnant women who attend antenatal care at primary and higher level health facilities.

**Methods:**

A community based study was conducted among 1108 nursing mothers. Data was collected during interviews using a structured questionnaire focused on socio-economic background, reproductive history, experience with antenatal HIV counselling and testing as well as types of health facility providing the services.

**Results:**

In all 91.0% of the women interviewed had attended antenatal care and 90.3% had been tested for HIV during their most recent pregnancy. Women who had their first antenatal checkup at primary health facilities were significantly more likely to be tested before 34 weeks of gestation (OR = 43.2, CI: 18.9-98.1). The reported HIV counselling provision was also higher at primary health facilities, where women in comparison with women attending higher level health facilities were nearly three or and four times more likely to receive pre-test (OR = 2.7; CI:2.1-3.5) and post-test counseling (OR = 4.0; CI: 2.3-6.8).

**Conclusions:**

The results suggest that antenatal HIV counseling and testing can be scaled up to primary heath facilities and that such scaling up may enhance early uptake of testing and provision of counseling.

## Background

Mother-to-child transmission (MTCT) is the main cause of HIV infection in children [1]. HIV counselling and testing for pregnant women is therefore considered a key factor for successful Prevention of MTCT (PMTCT) [2-4]. HIV counseling and testing is usually integrated with antenatal care at different levels of the health care system. However, the quality and uptake of the service varies, and it has been documented that even when HIV testing is offered as a part of antenatal care, many women are still tested for the first time only as they go into labor and thus do not get the full benefit from the PMTCT program [5,6].

In Asia, the HIV prevalence among pregnant women is 1-2% [5,7,8], which is low in comparison with sub-Saharan Africa, where prevalence rates of 5 to 37% have been reported [3,9]. PMTCT is considered an effective means to address this segment of the HIV epidemic and a number of PMTCT programs have been implemented worldwide during the past decade. The uptake of PMTCT services, however, varies greatly. For instance in India, in a recent report from a facility-based study, 96% of pregnant women said that they had been tested for HIV [6] while another community-based study from rural India documents that only 3% of pregnant women were tested for HIV [8]. The corresponding figures for Thailand and Hongkong were 93% [10] and 77% [11], respectively. Inaccessibility or lack of antenatal services as well as limited information on PMTCT are reported to be the main factors hindering antenatal HIV testing [1,6,12]. In addition, HIV related stigma and discrimination prevent many pregnant women from being tested for HIV [6,8,9,13]. With regard to the health care system, the health staff's attitude and sensitivity to the women's fear of stigmatization are crucial for successful implementation of PMTCT [1,8,14,15]. However, there is a lack of studies examining the timing of uptake of HIV testing and counseling provision at different levels of health facilities (HFs) of the health care system.

In Vietnam, the HIV prevalence among pregnant women is reported to be 0.37% [16]. However, this figure is under-estimated and covers only 16% of the real number of HIV-infected pregnant women [17]. PMTCT has become a priority for the government, which has launched a campaign against HIV/AIDS with the goal to offer HIV counselling and testing to 90% of pregnant women and to provide prophylactic interventions to 100% of those testing HIV-positive [5]. To reach this goal, PMTCT services have, with support from the government and international donors, been scaled up in a few selected pilot sites which are considered to be severely affected by the HIV epidemic. In these sites, the PMTCT services have been integrated into antenatal services at different levels of HFs. According to the PMTCT guidelines, which have been implemented in the pilot sites, pregnant women should be offered HIV counseling and testing at first antenatal care visit. At primary HFs, pre-test counselling, rapid HIV testing and post-test counselling are made available. If a woman is found to be HIV positive, she is referred to a higher level HF, where the full PMTCT program, including HIV counseling and testing and antiretroviral treatment for both the mother and her infant is provided free of charge. Early HIV testing among pregnant women is a challenge in Vietnam where until recently the policy was to provide routine HIV testing at labor. This trend is reflected in a substantial proportion (50-70%) of HIV positive pregnant women receiving their HIV diagnosis at labor [5,15,18].

The expansion of the PMTCT services to lower level HFs is recent and an assessment of the uptake of HIV testing at different levels of HFs as well as of the timing of the testing is needed to guide further planning of such programs. The uptake of HIV testing and counseling was measured by the frequency of women who were tested for HIV and provided counseling during pregnancy. This paper reports the results of a community-based approach investigating the uptake of HIV testing among pregnant women in a pilot site where PMTCT services have been implemented at primary HFs (commune health stations). It further describes the socio-economic characteristics of women who were tested at primary and higher level (district and provincial) HFs, and describes timing of testing and the provision of counseling services at different sites. These results provide a basis to discuss possibilities for the further scaling-up of PMTCT services in Vietnam.

## Methods

### Study site

The study was conducted in Quang Ninh, a coastal province in northern Vietnam. Quang Ninh, with an HIV prevalence among pregnant women of 1% [5], is one of the provinces in Vietnam hardest hit by the HIV epidemic.

In 2004, a PMTCT pilot program was implemented in three urban areas in Quang Ninh: Ha Long city, Bai Chay town and Uong Bi town. In these sites, HIV counseling and testing has been made available at primary HFs and at higher levels of HFs (secondary and tertiary levels). According to the PMTCT guidelines, pregnant women should be offered HIV testing at the first antenatal visit. If the pregnant woman has not been tested for HIV or is unaware of her status, she should be offered HIV testing free of charge at the time of delivery [5].

Ha Long city with surrounding communes was selected as study setting. In this area, antenatal care is available at all levels of HFs including 20 commune health stations, two secondary HFs (Bai Chay Hospital and Center of Maternal and Child Health Care) and one tertiary HF (the General Provincial Hospital). PMTCT has since 2004 been available at all commune health stations and at the General Provincial Hospital [5], whereas HIV testing is not supported by the PMTCT programme at secondary HFs. If a woman is detected HIV positive at one of the primary HFs, she is referred to the General Provincial Hospital for further treatment and care. HIV counselling and testing is additionally offered at several free standing centers.

### Study population

**Figure 1:**
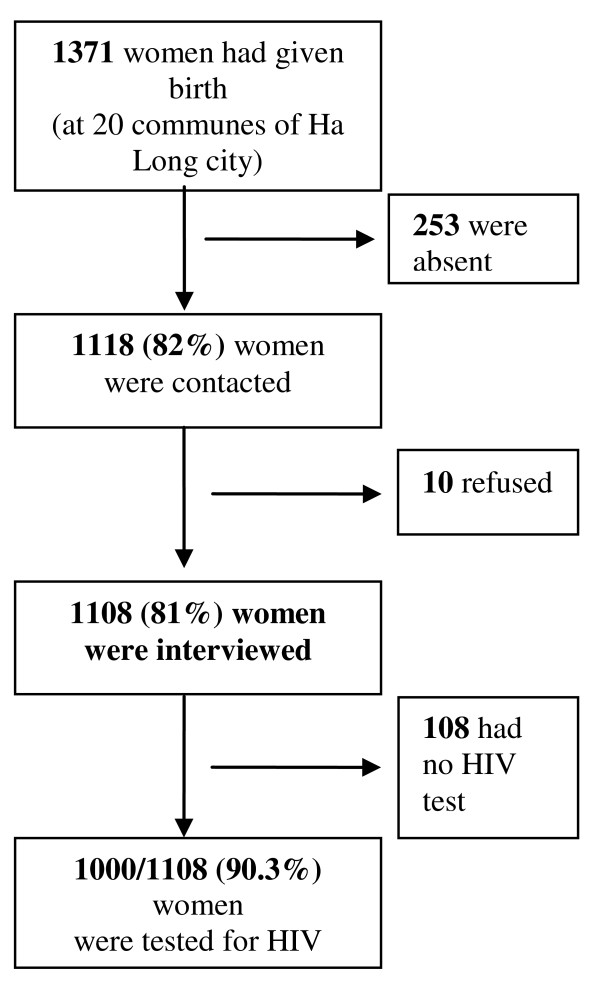
**Study population in relation to antenatal care and HIV testing**. This figure describes the process of selecting study population.

The study population comprised women living in Ha Long city who had delivered between January and June 2007. Ha Long city is divided into 20 communes, each of which keeps a "birth registration book" where the name and age of the women, together with the name and date of birth of their children, are recorded. The register does not, however, provide detailed addresses of the women; therefore, in order to identify women who had recently delivered, the researchers established contact with "population collaborators" who were responsible for birth registration at the commune level. Out of 1371 eligible women, 253 women were not at home when a visit was attempted; they were either spending the post-partum period at their mother's houses, were at work or were absent for other reasons. Of the remaining 1118 women, 1108 agreed to participate in the study (Figure 1).

### Data collection

A structured questionnaire was used to collect information about socio-economic background, reproductive history and experience with HIV counseling and testing and knowledge and awareness of PMTCT. Three research assistants performed the interviews, together with the population collaborators at commune level.

### Data analysis

Data were entered using the software EPIDATA and exported to SPSS for Windows, version 15.0 for analysis. The main outcomes variables of this study were (1) types of HF where women were tested for HIV; (2) time of the first antenatal HIV test; and (3) provision of HIV counselling.

Firstly, the socio-economic characteristics of the study population are described in relation to uptake of first antenatal HIV testing. Chi-square testing was applied to examine how socio-economic characteristics differed among women who were tested early, tested late or not tested (Table 1).

**Table 1:** Socio-economic characteristics in relation to uptake of first antenatal HIV test (tested early, tested late and un-tested).

Socio-economic characteristics of the women	Total N = 1108	The uptake of the first HIV test n (%) N = 1108			p-value
			
		*Tested early* * < = 34 wks*	*Tested late* * > 34 wks*	*Un-tested*	
		*781 (70.5)*	*219 (19.8)*	*108 (9.7)*	
**Age (mean of age = 28.7)**					
15-24	300 (27.0)	217 (72.3)	53 (17.7)	30 (10.0)	**p = 0.70**
25-34	672 (60.7)	467 (69.5)	142 (21.1)	63 (9.4)	
35-49	136 (12.3)	97 (71.3)	24 (17.6)	15 (11.0)	
**Number of children**					
1	563 (50.9)	424(75.3)	101 (17.9)	38 (6.7)	**p < 0.001**
2	512 (46.1)	338 (66.0)	107 (20.9)	67 (13.1)	
3 - 4	33 (3.0)	19 (57.6)	11 (33.3)	3 (9.1)	
**Education levels**					
Primary school	59 (5.3)	34 (57.6)	15 (25.4)	10 (16.9)	**p = 0.01**
Secondary school	332 (30.0)	228 (68.7)	60 (18.1)	44 (13.3)	
High school	344 (31.0)	258 (75.0)	61 (17.7)	25 (7.3)	
College/University	373 (33.7)	261 (70.0)	83 (22.3)	29 (7.8)	
**Occupation**					
Housewife/Unemployed	431 (38.9)	312 (72.4)	78 (18.1)	41 (9.5)	**p = 0.16**
Farmer/seasonal work	89 (8.0)	61 (68.5)	11 (12.4)	17 (19.1)	
Government staff/workers	352 (31.8)	251 (71.3)	74 (21.0)	27 (7.7)	
Business/Others jobs	236 (21.3)	157 (66.5)	56 (23.7)	23 (9.7)	
**Residence**					
Urban area	300 (27.1)	216 (72.0)	75 (25.0)	9 (3.0)	**p <0.0001**
Semi-urban area	395 (35.6)	304 (77.0)	74 (18.7)	17 (4.3)	
Remote rural area	413 (37.3)	261 (63.2)	70 (16.9)	82 (19.9)	
**Monthly income**					
< 1.5 (million VND)	254 (23.0)	175 (68.6)	47 (18.4)	33 (12.9)	**p = 0.39**
1.5 - 2.5	256 (23.1)	181 (70.4)	49 (19.1)	27 (10.5)	
> 2.5 - 3.5	333 (30.1)	232 (69.7)	73 (21.9)	28 (8.4)	
> 3.5	264 (23.8)	193 (73.4)	50 (19.0)	20 (7.6)	

Secondly, the women's socio-economic characteristics and their gestational age at first antenatal care visit were studied in relation to types of HF where the first HIV test was performed. Crude odds ratios (OR) were calculated using "Types of HF for the first HIV test" as dependent variables and the socio-economic characteristics and gestational age at first antenatal care visits as independent variables (Table 2). To adjust for the potential confounding effect of socio-economic characteristics, backward stepwise logistic regression was performed, where the variables found to be significant at a p < 0.05 level (educational level, occupation, residence and monthly income) were included in the final model [19].

**Table 2:** Socio-economic characteristics and gestational age of the first antenatal care visit among women having their first antenatal HIV test at primary and at higher level health facilities (HF)

Characteristics of the women	Types of HF for the first HIV test N = 1000		P values	Primary vs higher level	
			
	*Primary level *n (%)	*Higher level* n (%)		*Crude OR*	Adjusted OR*
	*434 (43.4)*	*566 (56.6)*			
**Age (mean of age) = 28.7)**			***p = 0.7***		
15-24	122 (44.6)	148 (55.4)		1.1 (0.8-1.4)	-
25-34	264 (43.3)	345 (56.7)		1	
35-49	48 (40.5)	73 (59.5)		0.9 (0.6-1.3)	
**Number of live children**			***p = 0.5***		
1	228 (43.4)	297 (56.6)		1,0 (0.8-1.3)	-
2	195 (43.8)	250 (56.2)		1	
3 - 4	11 (36.7)	19 (63.3)		0.7 (0.3-1.4)	
**Education levels**			***p < 0.0001***		
Primary school	29 (59.2)	20 (40.8)		2.9 (1.6-5.3)	1.7 (0.9-3.4)
Secondary school	151 (52.1)	137 (47.9)		2.1 (1.6-3.0)	1.5 (1.0-2.1)
High school	138 (43.6)	181 (56.4)		1.5 (1.1-2.1)	1.2 (0.9-1.7)
College/University	116 (33.7)	228 (66.3)		1	1
**Occupation**			***p < 0.0001***		
Housewife/Unemployed	204 (52.3)	186 (47.7)		2.4 (1.7-3.2)	1.7 (1.2-2.4)
Farmer/seasonal work	37 (51.4)	35 (48.6)		2.3 (1.4-3.8)	1.4 (0.8-2.5)
Government staff/workers	103 (31.7)	222 (68.3)		1	1
Business/Others jobs	90 (42.3)	123 (57.7)		1.6 (1.1-2.3)	1.4 (0.8-2.1)
**Residence**			***p < 0.0001***		
Urban area	97 (33.3)	194 (66.7)		1	1
Semi-urban area	168 (44.4)	210 (55.6)		1.6 (1.2 - 2.2)	1.7 (1.2-2.4)
Remote rural area	169 (51.1)	162 (48.9)		2.1 (1.5-2.9)	2.0 (1.4-2.8)
**Monthly income**			***p < 0.0001***		
< 1.5 (million VND)	125 (57.2)	97 (42.8)		2.4 (1.7-3.4)	1.7 (1.2-2.6)
1.5 - 2.5	115 (50.0)	115 (50.0)		1.8 (1.3-2.6)	1.5 (1.0-2.1)
> 2.5 - 3.5	111 (35.7)	194 (64.3)		1	1
> 3.5	84 (34.2)	159 (65.8)		0.9 (0.7-1.3)	1.1 (0.8-1.6)
**Time of first ANC visit**					
< = 34 weeks	385 (43.3)	505 (56.7)	***p = 0.8***	1	-
> 34 weeks	49 (44.5)	61 (55.5)		1.1(0.7-1.8)	

* Adjusted for the effect of educational level, occupation, residence and monthly income.

Thirdly, the relation between type of health facility and provision of HIV testing result is described among women who had their first HIV test before 34 weeks and women who had their first HIV test after 34 weeks gestation. To describe the association between timing of first HIV test and types of HF for first HIV test as well as the association between timing of first HIV test and provision of HIV information at first antenatal care visit, crude ORs were calculated using "Types of HF for first HIV test" and "The provision of HIV information" as independent variables while "Timing of first HIV test " was included as dependent variable (Table 3).

**Table 3:** Type of health facility (HF) and provision of HIV information at the first antenatal care visit among women who had their first HIV test before 34 weeks and after 34 weeks gestation.

	Timing of first HIV test		< 34 weeks vs >34 weeks Crude ORs
		
	*Tested < 34 weeks *n (%)	*Tested >34 weeks *n (%)	
***Types of HF for HIV test (N = 1000)***	*781 (78.1)*	*219 (21.9)*	
Primary HFs	428 (98.6)	6 (1.4)	43.2 (18.9.-98.1)
Higher level HFs	353 (62.4)	213 (37.6)	1
***The provision of HIV information*** at the 1^st ^antenatal care visit (N = 913*)	*725 (79.5)*	*188 (20.5)*	
No	483 (73.5)	174 (26.5)	6.2 (3.5 - 11.0)
Yes	242 (94.5)	14 (5.5)	1

* Number of the women who had HIV test and antenatal care during their pregnancies

Finally, to examine the difference in the provision of HIV counselling at different HFs, ORs were calculated where "Provision of HIV counselling (pre-test and post-test counselling) were included as dependent variables and "Types of HF" as independent variable (Table 4).

**Table 4:** Provision of HIV counselling (pre-test and post-test) at primary and higher level health facilities (HFs).

**Types of HFs**	Pre-test counseling (N = 997*)			Post-test counseling (N = 814**)		
		
	***Yes*** ***n (%)***	***No*** ***n (%)***	***Yes vs no*** ***Crude OR***	***Yes*** ***n (%)***	***No*** ***n (%)***	***Yes vs no*** ***Crude OR***
Primary HFs	225 (51.8)	209 (48.2)	2.7 (2.1-3.5)	57 (14.8)	329 (85.2)	4.0 (2.3-.6.8)
Higher level HFs	160 (28.4)	403 (71.6)	1	18 (4.2)	410 (95.8)	1

* Cases of missing data: 3

** No application for 184 women who did not come back for getting test results, and 2 cases of missing data.

All crude and adjusted ORs in the Tables in this article were calculated with 95% confidence intervals (CI).

### Ethical considerations

Ethical approvals were obtained from the Central Committee for Biomedical Research in Denmark, and from the Scientific Committee, General Office of Population and Family Planning, Ministry of Health, Vietnam. It was stressed that participation in the study was voluntary, and written informed consent was obtained before interviewing began. The field study was done with the permission of local authorities.

## Results

### Socio-economic characteristics and the uptake of HIV test

Table 1 shows the women's socio-economic characteristics in relation to their uptake of first HIV test. The age of the women ranged from 15 to 49 years, with a mean age of 28.2 years. Sixty percent of the women were aged 25-34 years. Fifty-one percent of the women had one child while only 3% had three or more children. Two thirds had high school education and higher. Nearly 40% of the women reported that they had no job. Twenty-seven percent resided in the center of city (Hon Gai), 36% in semi-urban areas (out-skirt of Hon Gai) and 37% in remote rural areas (Bai Chay). Nearly half of the women had a monthly income lower than 2.5 million VND (around 130 USD).

With regard to the first HIV testing, 90.3% had at least one HIV test during pregnancy. Four percent stated that they had not been tested for HIV, whereas 6% did not know whether they had been tested or not. Among the tested women, 43% had been tested at a primary HF and 78% were tested before or at 34 weeks of gestation.

### HIV testing at different levels of health facilities

Table 2 summarizes the associations between the women's socio-economic characteristics and the type of HFs where they had their first antenatal HIV test. Women of low education were more likely to have had their first HIV test at a primary HF in comparison with women who had a college or university education. Likewise, women who had unstable jobs, were living in semi-urban areas or had an income below 2.5 million VND were more likely to be tested at a primary HF when compared to government staff/workers, women living in urban areas and women with an income of 2.5-3.5 million VND, respectively. The time of the first antenatal care visit did not differ between women who had their first antenatal HIV test at primary level HFs and women who were tested at higher level health facilities. These associations were slightly less significant in the adjusted analysis.

### Early testing and provision of counselling

The associations between type of health facility and provision of HIV information at the first antenatal care visit among women who had their first HIV test before 34 weeks and after 34 weeks gestation are summarized in Table 3. Women who had been tested at primary HFs were in comparison with women who were tested at higher level HFs more likely to be tested for HIV before 34 weeks of gestation (OR = 43.2; CI: 18.9-98.1). More over, women who had received information on HIV testing at their first antenatal care visit were more likely to have had an HIV test early than was the group of women who had not received any information (OR = 6.2; CI: 3.5-11.0).

With regard to counselling, the proportions of women who reported that they had been provided with pre-test and post-test counselling were low (38.6% and 7.5%). However, women who had attended antenatal care at primary level HFs had, in comparison with women who had attended care at higher level HFs, significantly more often received pre-test (OR = 2.7; CI: 2.1-3.5) and post-test counseling (OR = 4.0; CI: 2.3-6.8) (Table 4).

## Discussion

Using a community-based rather than a facility-based approach in this study, we found that 90% of the pregnant women had been tested for HIV. Early uptake of the HIV test was more common among women who had attended antenatal care at a primary HF. Likewise, the provision of HIV counselling was also reported to be higher at primary HFs, where more women had received pre- and post-test counselling.

The study population comprised women from Ha Long city in Quang Ninh province, an area in which PMTCT has been widely implemented with support from foreign and international donor agencies. The findings therefore cannot be generalized to Vietnam as a whole since the PMTCT program is not implemented in the same way in all provinces. Yet the study does offer insights into the dynamics of a pilot site where the PMTCT services have been scaled up to community level and the findings may thus be relevant for a more general expansion of PMTCT services in the whole country. Concerning the representativeness of the study population, we were not able to obtain background characteristics of the women who did not participate in the study and were therefore not able to assess whether they differed systematically from the women who participated in the study. However, since 81% of the eligible women were included in the study, it may be argued that the findings represent the vast majority of women who had recently delivered in the study setting. Regarding the internal validity of the study, the information about HIV testing was obtained from the women through questionnaire interviews. The women's answers were not checked against any formal registration of the gestational age at which the women were tested. This lack of cross checking may have affected the results. The uptake of antenatal HIV testing was high; 90% had been tested for HIV, either at the time of antenatal care or at the time of labor. The high rate of HIV testing found in our study is in line with experiences from Thailand, where 93% of pregnant women attending antenatal care were tested for HIV [10]. However, a facility-based study in the neighboring province, Hai Phong in 2005 showed that only 53% of the pregnant women had been tested for HIV [20]. The studies from Hai Phong province (Vietnam) and Thailand were both facility-based, whereas our study was community-based. One of the advantages of a community-based design is that it covers women regardless of whether or not they have had contact with a HF during their pregnancy and labor and may thus, in comparison with studies which rely on a facility-based design, provide a more trustworthy picture of the acceptance of HIV testing in a society [21].

This study showed that the early uptake of HIV testing and provision of counseling differed depending on HF level. Early HIV testing was more common among women who had had their first antenatal visit at a primary HF, where the women had also more often been provided with pre- and post-test counselling. At the higher level HFs, women were generally tested later in their pregnancy and were not provided counselling. However, when evaluating the HIV testing services offered at different HF levels, the timing of the antenatal visit at the different levels should be taken into consideration. If 'the first antenatal' visit at a higher level facility is actually after 34 weeks of gestation or during labor, while women are attending primary facilities for their first visits for 'normal' antenatal care, this would affect timing of HIV testing and the motivation/ability of healthcare workers to tackle HIV testing. However, no significant difference of the time of the first antenatal care visit was found between primary level and higher level of HFs (Table 2). In addition women who were tested at lower level HFs were more likely to have received counseling in relation to the testing. A likely explanation for the earlier uptake of HIV testing as well as for the higher proportion of women receiving counseling at lower level HFs may be that health staff working in higher level HFs often are preoccupied with many different assignments and do not have sufficient time to spend on HIV counselling and testing; further, they are often unable to offer privacy during counselling [5,15,22]. In contrast, primary level HFs have better conditions in terms of both time and space for providing counselling and HIV testing for pregnant women [5]. Hence, the antenatal care nurses who worked at primary level HFs were apparently in a better position to promote HIV testing. This assumption is supported by a number of in-depth interviews showing that pregnant women in Ha Long found the health staff at primary level HFs skilled in tailoring the HIV information and counselling to address the individual women's circumstances and concerns [23].

Early and voluntary HIV testing is increasingly being challenged. Due to increased availability of antiretroviral treatment, policies on HIV testing have shifted towards routine testing for HIV as a part of antenatal care [24-27]. A recent community-based study from Hanoi has documented that 85% of pregnant women were tested late and received inadequate counseling due to a lack of PMTCT services at the commune level [21]. Hence, many Vietnamese women who are tested for HIV during pregnancy are in a position where they do not get the full benefit of the PMTCT program. Against this background it is encouraging that nearly 71% of the women in our study had been tested for HIV before or at the 34th week of gestation. This high rate of early HIV testing suggests that the antenatal care program in Quang Ninh province is functioning well, an assumption that is supported by the fact that 82% of pregnant women attend antenatal care (90% at urban sites and 80% at rural sites), and that the vast majority come for antenatal care during the first trimester [5]. Thus, excellent conditions exist for an efficient PMTCT service which, in the setting studied, has been backed up by massive investments in both the quality and the quantity of PMTCT.

HIV counselling and testing late or during the time of labor has been advocated to be a rational way to increase PMTCT uptake [25]. However, HIV testing at the time of labor should be treated as the last resort for prevention of MTCT, because the women then miss the opportunity to receive the full prophylactic regime as well as other PMTCT services [5,6]. Moreover, being confronted with a positive HIV result is associated with great distress [22,23] and labor is not the optimal time for conveying such information [23,28]. One way to avoid these problems is to offer women HIV testing at primary HFs, when they have their first antenatal checkup.

A successful scale-up of HIV counseling and testing to lower level HFs has been documented in this study. Other studies in Vietnam have shown that the implementation of HIV testing at lower level HFs or by outreach workers may be an effective way to scale up PMTCT [14,29]. This approach may especially apply for settings in which the lack of HIV testing at commune level is one of the main reasons for poor quality PMTCT [21,30]. Moreover, the results of this study are in line with studies in other countries which have shown that the provision of HIV counseling and testing at community level may increase access to the service for vulnerable rural women and place them in a position where they can access and benefit from PMTCT programs [4,8,14,29].

## Conclusions

The present study documents that HIV testing and counseling for pregnant women can be enhanced if the PMTCT service is incorporated into the antenatal care program at primary level HFs. Although it may be questioned whether the present study can be generalized to settings where less massive investment in PMTCT is made, it is argued that the findings may serve as an important inspiration for the future expansion of PMTCT service in Vietnam as well as in other low income countries. To shed further light on the uptake of PMTCT in resource poor settings it is recommend that additional studies are performed in sites where other conditions for PMTCT prevail.

## List of abbreviations

HF(s): Health facility(ies); MTCT: Mother to child transmission of HIV; PMTCT: Prevention of mother to child transmission of HIV

## Competing interests

The authors declare that they have no competing interests.

## Authors' contributions

NTTH participated in designing the study, conducted the data collection, analyzed the data and drafted the manuscript. VR participated in designing the study, in outlining the manuscript, and provided critical comments through the writing process. TG participated in designing the study, in outlining the manuscript and provided critical comments for finalizing the paper.

All authors read and approved the final manuscript.

## Pre-publication history

The pre-publication history for this paper can be accessed here:

http://www.biomedcentral.com/1472-6963/11/29/prepub
